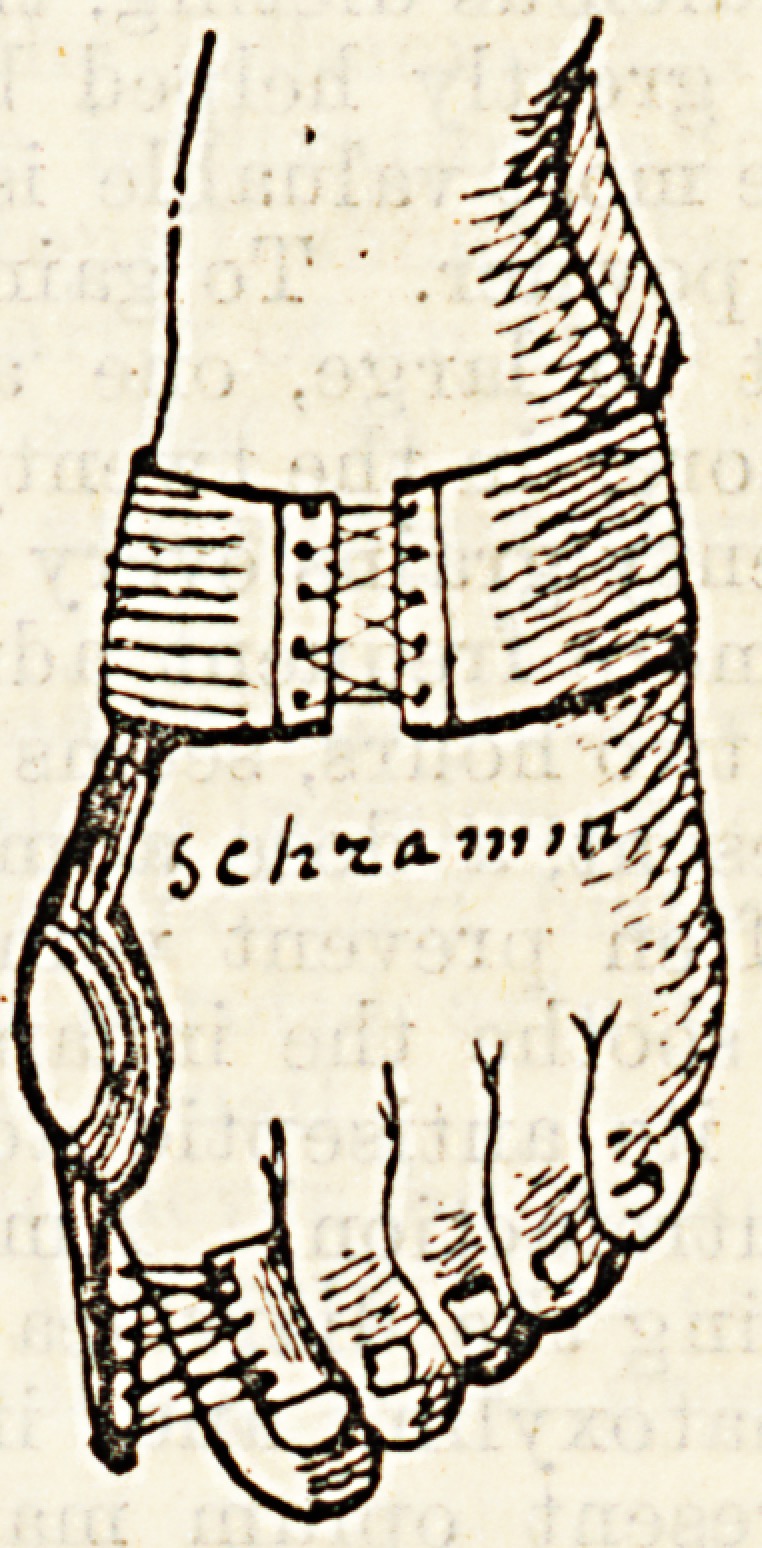# Treatment of Hammer Toe

**Published:** 1893-09-09

**Authors:** 


### The Hospital Clinic.

[*The Editor will be glad to receive offers of co-operation and contributions from members of the profession. All letters should be addressed to* The Editor, The Lodge, Porchester Square, London, W.]

### ROYAL ORTHOPÆDIC HOSPITAL.

### Treatment of Hammer Toe.

This affection consists of a flexion of the toe, generally the second, so that the pressure falls on the very end of the toe, which is flattened and expanded thereby. In some cases it gives rise to no trouble, but in most, sooner or later, a sore developes on the sharp angle formed by the bending of the toe. This usually gives rise to a great deal of trouble. Hammer toe is often a family complaint, and it is by no means unusual to find several members suffering from it. Its successful treatment is not found to present much difficulty as a rule. The tense structures on the under surface of the toe, which hold it in its deformed position, are divided subcutaneously. As a rule it is only necessary to divide the lateral ligaments of the joint between the first and second phalanges, but sometimes the flexor tendon has to be divided also. After the operation the wound is dressed with a little lint, and the foot is fixed with a metal splint applied to the sole so that its end projects beyond the affected toe, which is fastened to it by a bandage in such a way as to straighten it. Every day the toe is stretched by manual force, and after a few days the wound being healed it is removed from the splint and placed on a sort of sandal, by means of which the further treatment is pursued.

The diagram shows one of the forms in use. It consists of a metal plate turned up around the heel so as to prevent it slipping forwards. In front there are some slots through which tapes can be drawn. These are passed over the toes and then tied beneath the sole plate so that they constantly exert a straightening effect on the toes. Some of the surgeons use a sandal which is soft and pliable except for a steel plate at the toes. After the toe has once been straightened, a boot is ordered large in size and broad at the sole. During the night it is directed that the sandal be worn for long after the deformity is nominally cured in order to prevent its return. The patient, too, is told firmly to extend the toe night and morning.

*Hallux Valgus.*—This is the name applied to the condition in which the great toe is forced outwards from its proper position so that it presses upon and usually overlies those next to it. It is regarded as being in many cases the result of wearing boots with too narrow toes. It probably may also be caused by wearing, especially during childhood, socks or stockings with pointed instead of square toes. An unpleasant complication of this deformity is the formation of a bursa on the inner side of the metatarsalphalangeal joint, which is liable to become inflamed and form a bunion. The condition is most usually treated by means of the spring mechanism shown in the diagram. This consists of a piece of a metal spring, which is fixed to the inner edge of the foot, and which runs up the side of the great toe carrying a loop by which the end of the toe is fixed to it. It thus constantly exerts a slight force on it tending to draw it back into its proper position, and at the same time protects it from injury. Many cases get well, or at any rate cease to give trouble when this instrument has been applied to them. Others, however, derive little or no benefit from it, and for them a more radical measure is sometimes used. The toe joint is excised. This is done by making an incision along the inner side of the joint. The soft tissues are then separated by means of a raspatory, the joint is divided with a knife, and the bone cut through just above with a bone-forceps. The wound is now sewn up, and the foot fixed on a splint with the toe in the line of the foot. With proper care in the after treatment a movable toe is obtained with entire cure of the deformity.

*In-growing Toe Nail.*—This is often the trouble that brings patients to the Royal Orthopædic Hospital. It is in most cases the result of wearing narrow-toed boots. The toes are thereby squeezed up together, the toe-nails are bent, and the flesh at the sides is pressed up over them. The complaint may be met with in every stage from slight discomfort present only on walking to that in which the nail is embedded in a mass of fungating granulations, which bleed on the slightest touch. The slighter cases are treated merely by reversing the conditions that give rise to the trouble. The patient is directed to wear a large boot with square toes, also that the nails be cut straight across or in a half moon shape, so that the nail is higher at the sides than at the middle, and that the centre of the nail be scraped so that it is thinned and rendered somewhat pliable. More severe cases are treated according to their severity. If there be no ulceration, a strip of thin silver is taken, and its edge at one end bent in a sharp angle. This is slipped under the side of the nail, and then the rest of the strip is carried over the flesh growing over the nail, and round the toe. In this way the nail is drawn out of the hollow in which it lies at the same time that the flesh is pushed off it. A strip of lint is sometimes used instead of the silver, but is not so efficient. A somewhat similar form of treatment is that carried out with a piece of cork. A semicircle is cut from the cork of a champagne bootle, it is applied to the nail with its concavity downwards. A narrow bandage is carried round the toe and cork both, and thus the flesh is pushed back from the edges of the toe. This takes place because the piece of cork is more sharply curved than the nail, and, therefore, when it is flattened out by the pressure of the bandage, it exerts pressure on the structures in contact with its edges. In the severer cases it is sometimes necessary to remove the nail, or following the American plan, to cut away the overhanging granulations, and at the same time the flesh on which they rest. This proceeding, if efficiently performed, is very successful, since the contraction of the scar that results constantly tends to drag the flesh from the side of the nail, and so prevent the recurrence of the deformity.

## Figures and Tables

**Figure f1:**
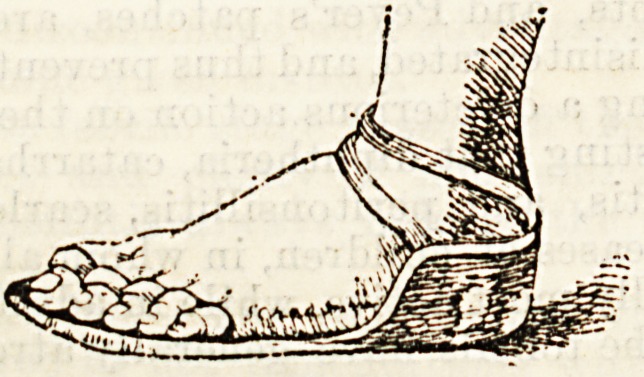


**Figure f2:**